# Quaternized chitosan/PVA/natural bioactive agent electrospun wound scaffolds: production, characterization, and investigation of release kinetics

**DOI:** 10.55730/1300-0527.3633

**Published:** 2023-10-16

**Authors:** Ezgi EREN BELGİN, Cankız Gizem DELİBALTA

**Affiliations:** 1 Department of Chemistry, Faculty of Science, Muğla Sıtkı Koçman University, Muğla, Turkiye; 2 Biomaterials and Tissue Engineering Laboratory, Muğla Sıtkı Koçman University Research Laboratories Center, Muğla, Turkiye

**Keywords:** Quaternized chitosan, electrospun scaffold, *Calendula officinalis*, wound dressing, release kinetics, extract loaded fiber

## Abstract

Quaternized chitosan (HTCC) was synthesized and characterized to increase chitosan solubility. Then HTCC was electrospun with poly (vinyl alcohol) (PVA) and prepared natural bioactive agent (*Calendula officinalis*) extract was loaded onto fibers for wound scaffold applications. Morphological, structural, and mechanical characterization of the produced wound scaffolds was performed and their in vitro bioactive component release behavior was investigated. As a result, it was observed that the degree of quaternization of chitosan was 0.89, and synthesized HTCC was soluble in acidic, basic, alkaline media and could be electrospun with PVA in the presence of a natural bioactive agent. The presence of HTCC increased Young’s modulus and the tensile strength of the PVA scaffolds, while the presence of bioactive extract caused a decrease in Young’s modulus and an increase in tensile strength. *Calendula officinalis* is released in a controlled and slow manner from the scaffolds within approximately 55 h. The release behavior was consistent with the Higuchi kinetic model. In this study, the effect of PVA cooperator on HTCC nanofiber production in the presence of a bioactive component was investigated for the first time. HTCC and *Calendula officinalis* extract were also used together for the first time in the composition of a fiber scaffold. The mechanical properties and release kinetics of these scaffolds were also investigated for the first time. According to the results, it is thought that the wound scaffolds produced have the potential to be used as a new treatment tool, especially for chronic wounds.

## 1. Introduction

Chitosan fibers are one of the most suitable polymers used in wound dressing compositions due to their properties such as biodegradability and biocompatibility [1–3]. However, chitosan fibers have disadvantages such as poor liquid absorption capacity, poor antibacterial activity, and difficult dissolution in water or organic solvents due to their high crystalline structure. Thus, chitosan fiber application areas are limited due to these disadvantages. On the other hand, HTCC has higher antibacterial activity, higher fluid absorption capacity, higher capacity to provide a moist environment to the wound, and low fiber compaction and adhesion in the wound [4–8]. Since HTCC dissolves in a wide pH range, unlike chitosan, it is easier to use and has wider application areas. Electrospinning of HTCC is much easier using a nonionogenic cooperator [9–13] like PVA. Thus, firstly HTCC is synthesized and characterized and then electrospun with a PVA cooperator in the study. During the electrospinning process, a bioactive material that is extracted from *Calendula officinalis* (*C. officinalis*) flowers was also loaded into the polymeric fibers. *C. officinalis*, which is known as the m**arigold** or c**alendula** and belongs to the family Compositae, is an annual herb that can thrive in many soil types [14,15], and is used in the study as a bioactive ingredient. In the literature, in vitro studies with alcohol extract of *C. officinalis* showing human fibroblasts [16–19] and an increase in proliferation and migration of keratinocytes grown in cultures have been reported [19]. In a study, the voltage values at which PVA/sodium alginate (SAlg) electrospun fibers were obtained were 15–20 kV in the presence of *C. officinalis* extract, and an increment in fiber diameters with extract addition was reported [20]. *C. officinalis* extract has been also reported to have positive healing effects on different types of wounds such as acute wounds, bu°rn wounds, and chronic wounds in the literature [17,19,21–23]. There are studies in the literature showing that *C. officinalis* is loaded onto several polymeric fibers such as polyurethane, polycaprolactone, chitosan, poly (ethylene oxide), hyperbranched polyglycerol, and PVA [24–27], but in the present study it is incorporated with HTCC/PVA electrospun fibers for the first time.

## 2. Materials and methods

### 2.1. Materials

Medium weight chitosan (Sigma Aldrich), PVA (87%–89% hydrolyzed, Sigma Aldrich), glycidyl trimethyl ammonium chloride (GTMAC, Sigma Aldrich), acetic acid (CH_3_COOH, Riedel-de Haen), sodium acetate (CH_3_COONa, Merck), sodium hydroxide (NaOH, Merck), hydrochloric acid (HCl, 37%, Merck), perchloric acid (HClO_4_, Isolab), potassium chromate (K_2_CrO_4_, Merck), silver nitrate (AgNO_3_, Sigma Aldrich), ethanol (C_2_H_6_O, Sigma Aldrich), glutaraldehyde (C_5_H_8_O_2_, 25%, Sigma Aldrich), phosphate buffered saline tablet (PBS, Sigma Aldrich), and *C. officinalis* flowers were commercially supplied and used without further purification.

### 2.2. Quaternized chitosan (HTCC) synthesis

**Figure 1:**
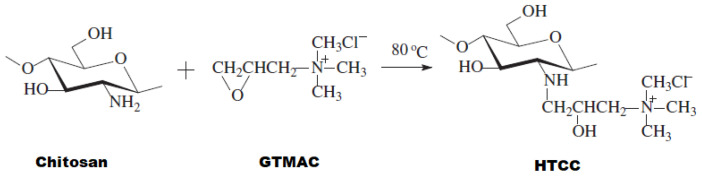
Synthesis reaction of HTCC.

The method applied for the HTCC synthesis was prepared by modifying the literature studies [28–30]. For HTCC synthesis 12.3 mmol of chitosan was dispersed in 30.0 mL of deionized (DI) water, 1.90 g of perchloric acid was added, and the chitosan was dissolved at room temperature. Then a solution of 98.8 mmol GTMAC was prepared with 30.0 mL of DI water. The chitosan solution was kept at 60 °C and GTMAC solution was added in three portions at 30-min intervals to enable the reaction. The reaction then continued at 80 °C for 8 h. The resulting turbid and yellowish reaction solution was poured into acetone and the product was precipitated. After the product obtained was washed three times with acetone, the white product was collected by filtration. Then the product was dissolved in DI water and precipitated again with acetone to obtain higher purity HTCC. Finally, the product was filtered and dried at 60 °C. The reaction is given in Figure 1.

### 2.3. Characterization of synthesized HTCC

#### 2.3.1. Determination of chitosan’s molecular weight

The exact molecular weight of the chitosan that is used for the synthesis of the HTCC was determined before starting the synthesis procedure. Briefly, chitosan solutions with different concentrations between 0.001 and 0.004 g mL^−1^ were prepared in CH_3_COONa (0.10 M)/CH_3_COOH (0.20 M) buffer solution (1:1 (v/v), pH 4.4) and the flow rates of the solvent (t_0_) and the chitosan solutions (t) were determined by Ubbelohde viscosimeter at 37 °C. The measurements were made three times and the average values were accepted as the flow rates. Then the relative viscosity (η_r_), specific viscosity (η_sp_), and reduced viscosity (η_red_) values were calculated using Equations (1)–(3) [31,32].


(1) $$\eta_{r}=\frac{t}{t_{0}}$$

(2) $$\eta_{sp}=\eta_{r}-1$$

(3) $$\eta_{red}=\frac{\eta_{sp}}{c}$$

Here C represents the concentration. After determination of the η_sp_ values the intrinsic viscosity [η] value of the chitosan was determined from the intersect of the plot of η_red_ with respect to concentration. Finally, chitosan average molecular weight was calculated by the Mark–Houwink equation, Equation (4) [33,34].


(4) $$[\eta ]=KM_{\nu }^{\alpha }$$

where M_v_ represents the molecular weight of the chitosan and K and α represent empiric constants for polymer–solvent pairs.

#### 2.3.2. Determination of the degree of quaternization

The degree of quaternization (DQ) of the synthesized HTCC was determined by titration with silver nitrate (AgNO_3_) solution in the presence of potassium chromate indicator. The chloride ions on HTCC were reacted with silver ions and the DQ value was calculated using Equation (5), where C (mol L^−1^) represents AgNO_3_ concentration, V (mL) represents AgNO_3_ volume, W (g) represents HTCC mass, M_1_ (g mol^−1^) represents glucosamine molar mass, and M_2_ (g mol^−1^) represents HTCC molar mass.


(5) $$DQ=\frac{CV/1000}{(CV/1000)+(W-(CVM_{2}/1000))/M_{1}}$$

Water solubility of the synthesized HTCC was investigated for pH 4.0, 5.0, 6.0, 7.0, and 8.0 values. For this, 10.0 mL of DI water was used as the solvent, the pH of the water was adjusted using 0.10 M NaOH or HCl solutions, and total dissolution of 30.0 mg mL^−1^ HTCC was accepted as soluble.

#### 2.3.3. Fourier transform infrared (FTIR) spectroscopy analysis

FTIR (Thermo Scientific Nicolet IS10) analysis was carried out for the synthesized HTCC and chitosan to verify the newly formed functional groups on HTCC. The samples were placed onto the attenuated total reflectance (ATR) crystal and pressed down using the swivel press to ensure optimal contact between the powder sample and the crystal. After sample placement, the analysis was carried out between 400 and 4000 cm^−1^ wave numbers.

### 2.4. Bioactive component (*C. officinalis)* extract preparation

*C. officinalis* flowers were washed with DI water, cut into small pieces, and dried at 40 °C for 72 h. Approximately 200 g of dried *C. officinalis* was incubated for 24 h in 3:7 (v/v) ethanol, then the solution was filtered, and the extraction step was performed three times. The total solution was dehydrated at 42 °C for 12 h using a rotary evaporator and dried at room temperature. The *C. officinalis* extract obtained was stored at −20 °C until used [35–37].

### 2.5. Electrospinning process

Firstly, 20% (w/v) HTCC solution at room temperature and 20% (w/v) PVA solution at 70 °C were prepared in DI water. Then the PVA and HTCC solutions were mixed at the desired ratios. *C. officinalis* extract was also added to the mixture at the desired ratio for the production of scaffolds that contain bioactive components. The mixtures were homogenized using an ultrasonic homogenizer.

Fibers were prepared using a uniaxial horizontal electrospinner (Inovenso Nanospinner). Firstly, the electrospinning solutions were drawn into a 5 mL disposable syringe with a capillary needle 1 mm in diameter. A capillary needle was placed in a syringe pump and connected to the positive terminal of the voltage source. The earth terminal was connected to the connector plate, which was coated with aluminum foil. The process parameters were optimized as follows: 18.0 kV voltage, 15.0 cm needle tip to rotor collector distance, 0.09 mL h^−1^ solution flow rate during the process, and fibers were collected on the aluminum foil.

One group of the produced scaffolds was then crosslinked using glutaraldehyde vapor for release studies since noncrosslinked scaffolds are highly soluble. The cross-linking procedure was carried out in a sealed vessel containing 0.25 M glutaraldehyde solution. The scaffolds were fastened on the cover of the vessel and then exposed to the glutaraldehyde vapor for 12 h.

The produced scaffold contents and the codes of the samples are given in Table 1.

### 2.6. Scaffold characterization

#### 2.6.1. Morphology

Scanning electron microscopy (SEM, Quanta 250) analysis was performed for the scaffold fiber morphology, fiber diameter, and homogeneity investigations.

#### 2.6.2. Mechanical properties

The mechanical properties of the scaffolds were investigated by dynamic mechanic analysis (DMA, TA – Instruments - Q800) method. The analysis was conducted at 37 °C.

#### 2.6.3. In vitro bioactive component release kinetics

Firstly, crosslinked *C. officinalis* loaded scaffolds (C-PVA/HTCC/CO) were cut to 2.0 × 2.0 cm size and transferred to a sealed vial containing 50.0 mL of pH 7.4 PBS solution for release kinetic studies. The vials were placed in an incubator shaker working at 200 rpm and 37 °C. Samples were taken from the vials in certain time intervals (0–62 h) and absorbance of the solution was read spectrophotometrically (UV-Vis, Thermo Scientific MultiSkan GO). UV-Vis scanning was performed in the range of 200–800 nm and absorbance values were recorded. The same procedure was also carried out for unloaded scaffolds to determine the net absorbance value of the extract by taking the difference of the absorbance values of loaded and unloaded scaffolds in each time interval. In order to calculate the amount of extract released from the scaffolds 20 standard solutions of *C. officinalis* extract were prepared between 5.0 × 10^−5^ and 25.0 × 10^−5^ concentration. UV-Vis scanning of the standard solutions was performed for 200–800 nm and the absorbance values obtained were plotted against the concentration of the standards to yield a calibration curve and equation. By using the calibration equation and UV-Vis data, the concentration and mass of *C. officinalis* released from the scaffolds were calculated. The *C. officinalis* release of scaffolds was presented in terms of percent cumulative release (CR), which is defined as the percentage ratio of the instantaneous amount of *C. officinalis* released at a certain time of incubation (C_t_) to the initial amount of *C. officinalis* loadings (C_i_), Equation (6).


(6) $$CR(\%)=\frac{C_{t}}{C_{i}}$$

After the determination of CR values, the data were fitted to linearized drug release kinetic models of zero order, first order, Hixson–Crowell, Higuchi, and Korsmeyer–Peppas. Regression coefficients were determined to obtain the best fit model for the data [36].

## 3. Results and discussion

### 3.1. Molecular weight of chitosan

The molecular weight of the chitosan used for the synthesis of HTCC determined by Ubbelohde viscosimeter studies was 262,409.6 g mol^−1^. In fact, the chitosan used in the study was ‘medium molecular weight’ and the molecular weight given by the manufacturer was 190–310,000 g mol^−1^. Therefore, the result obtained was within the expected range.

### 3.2. The degree of quaternization (DQ) of HTCC

The DQ value, which represents substituted quaternary ammonium salt groups of the synthesized HTCC, as determined by titration was 0.89. The determined DQ value was higher than the DQ value given in Alipour et al.’s [28] study in which a similar synthesis method was used and the DQ value was 0.789. This result confirmed the substitution of amino groups by the quaternary ammonium salt groups in chitosan.

### 3.3. The water solubility of HTCC

HTCC was totally dissolved in all studied acidic, neutral, and basic solvent media with pH 4.0, 5.0, 6.0, 7.0, and 8.0. This was an expected result due to quaternary ammonium groups of the HTCC with positive charges. The 30 mg mL^−1^ HTCC samples were completely dissolved in approximately 10–14 min under 250 rpm stirring at room temperature. The fastest dissolution was observed in neutral media with a 10 min 36 s dissolution time.

### 3.4. FTIR analysis of HTCC

**Figure 2:**
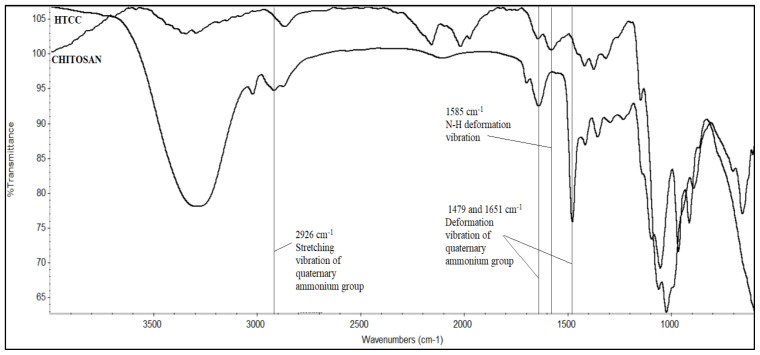
FTIR spectrum of chitosan and HTCC.

The FTIR spectrums of the chitosan used for the HTCC synthesis and the synthesized HTCC are given in Figure 2.

The N–H deformation vibration peak of chitosan seen at 1585 cm^−1^ was absent in the HTCC spectrum. Instead, three new absorption peaks at 1479 cm^−1^, 1652 cm^−1^, and 2926 cm^−1^ were observed. These peaks are attributed to the deformation vibration peak and the stretching vibration peak of –CH_3_ in the quaternary ammonium group of HTCC. Thus, the –NH_2_ groups of chitosan are replaced with the –CH_2_CH(OH)CH_2_N^+^(CH_3_)_3_Cl^−^ group, which demonstrates HTCC (Figure 1) [38–40].

### 3.5. SEM analysis of the scaffolds

**Figure 3:**
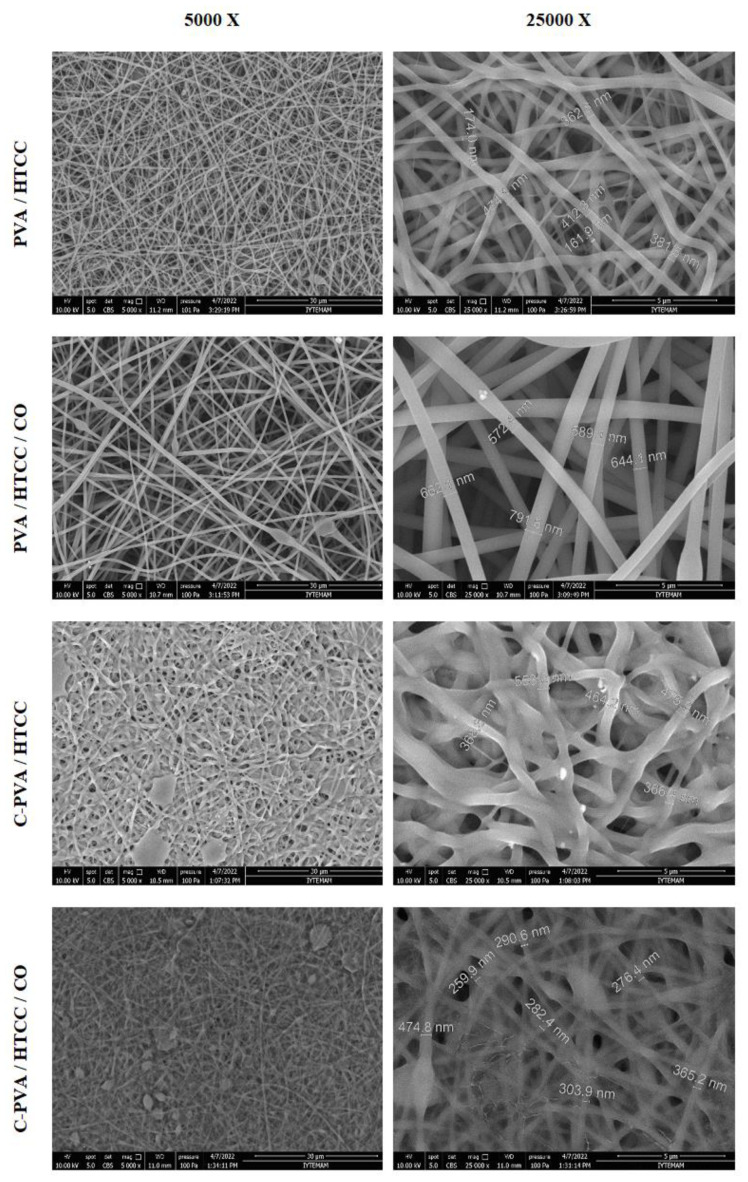
SEM nanographs of the scaffolds.

SEM micrographs of PVA, PVA/HTCC, and PVA/HTCC/C scaffolds with 5000× and 25,000× magnitude are given in Figure 3 for both crosslinked and noncrosslinked scaffolds.

The morphology of the electrospun nanofibers was uniform for noncrosslinked fibers with/without extract loading. After the crosslinking reaction with glutaraldehyde vapor, the fibers were crosslinked and intertwined. The morphology of the fibers was corrupted and especially the intersection points of the fibers were fused. This was due to the high-water solubility of the fibers dissolved with the water content of the exposed glutaraldehyde vapor during the crosslinking reaction. This result is expected in the crosslinking of water-soluble polymeric fibers with glutaraldehyde vapor and is also encountered in the literature [41].

The average, minimum, and maximum diameters of the scaffolds were determined by taking measurements at 50 points for each sample via the software ImageJ. The results are given in Table 2. When the effect of extract loading on fiber diameter is considered it is seen that the fiber diameter was increased by approximately 60% with extract loading. The viscosity of the electrospinning solution is one of the parameters that affects nanofiber diameter and the viscosity of a solution shows a linear relationship between its concentration and nanofiber diameter. It is expected that the viscosity of the electrospinning solution increases when *C. officinalis* extract is added to the solution. Thus, the viscosity increment leads to diameter increments of the nanofibers as reported in the literature [42,43]. After crosslinking reactions, the average fiber diameter was expected to decrease since the polymer chains got closer and the polymer shrunk. This shrinkage effect was observed for extract loaded fibers with approximately 51% shrinkage but the diameter was increased for nonloaded fibers after crosslinking instead of decreasing. The fused fibers dissolved with the water content of glutaraldehyde vapor caused the fiber diameter to appear higher at the surface of the scaffolds.

### 3.6. DMA analysis of the scaffolds

**Figure 4:**
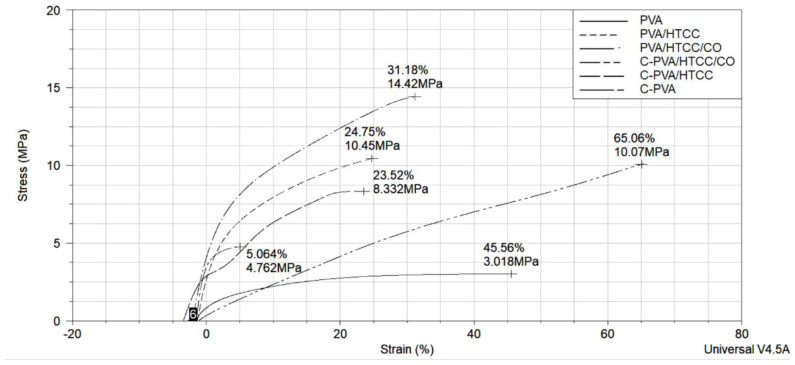
Stress–strain curve of the crosslinked and noncrosslinked scaffolds.

The stress–strain curves of the noncrosslinked and crosslinked scaffolds are given in Figure 4. The initial Young’s modulus of the scaffolds is also determined by calculating the slope of the linear region of the stress–strain curves. The tensile strength, elongation at break, and Young’s modulus values of the scaffolds at 37 °C are summarized in Table 3.

When the mechanical properties of the noncrosslinked dressings were evaluated, it was observed that the presence of HTCC partner increased the Young’s modulus by approximately 388% and the tensile strength by approximately 246% of the PVA scaffolds. Loading *C. officinalis* extract onto PVA:HTCC scaffolds caused a decrease of approximately 4.38% in Young’s modulus and an increase of approximately 37.9% in tensile strength. After crosslinking, Young’s modulus of the PVA:HTCC scaffolds decreased by approximately 62.7%, while the tensile strength decreased by approximately 20.2%. Loading *C. officinalis* extract onto crosslinked PVA:HTCC scaffolds also caused an approximately 57.2% decrease in Young’s modulus and an approximately 20.9% increase in tensile strength. When all mechanical test results were evaluated, it was understood that HTCC cooperator increased the stiffness and strength of the PVA fibers. Extract loading also increased the strength of the fibers while decreasing stiffness. On the other hand, no significant correlation was observed between the mechanical values obtained after the crosslinking process. It is thought that the reason is the fiber fusion on the surface of the scaffolds (as seen in the SEM images) during the crosslinking process with glutaraldehyde vapor. The inner parts of the scaffolds preserve their fibrous structure while the surface fibers are fused, which leads to heterogeneous behavior in mechanical properties.

### 3.7. In vitro extract release kinetics of the scaffolds

**Figure 5:**
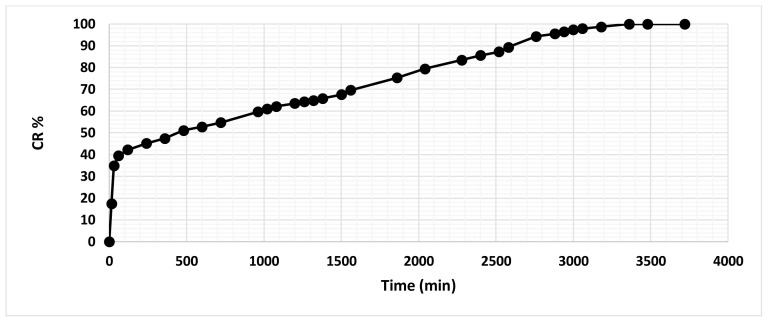
CR% values of the scaffolds with respect to time.

Within the scope of the in vitro extract release kinetic studies, no overlaying absorbance peak was found in the UV spectra of unloaded scaffolds within 446–450 nm, where the *C. officinalis* absorbance peak was observed. Thus, it was accepted that the absorbance values read in the UV spectrum for loaded scaffolds belonged to *C. officinalis* extract only. The plot of the calculated CR% values of the scaffolds with respect to time is given in Figure 5.

In vitro release analyses have shown that approximately 40% of the active substance loaded on the wound dressings is burst released in the first 60 min. This quick initial release might be due to the presence of the active substance on the surface of the fibers. Electrospun fibers have high surface due to their nature with larger amounts of surface associated active substance [44,45]. After the first 60 min, the active substance is released in a slow manner as a result of the conformational change and biodegradation of the polymer chains in the form of a fibrous structure due to swelling. After 3180 min, the extract concentration in the PBS solution did not increase significantly, which indicates that all of the *C. officinalis* content was released.

**Figure 6:**
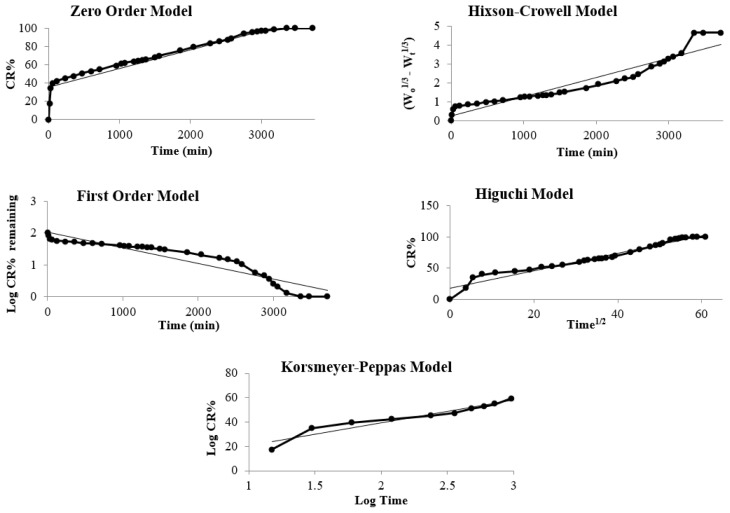
Zero-order, first-order, Higuchi, Hixson–Crowell, and Korsmeyer–Peppas kinetic models plot for release from scaffolds.

The kinetic model of *C. officinalis* release of scaffolds was studied by fitting the experimental data to zero-order, first-order, Higuchi, Hixson–Crowell, and Korsmeyer–Peppas models [46,47] and the model plots are given in Figure 6. The calculated kinetic parameters are given in Table 4.

According to the regression coefficients in Table 4, the release kinetics of the scaffolds best fitted the Higuchi kinetic model. The Higuchi equation is based on several assumptions, i.e. the initial concentration of the drug is higher than the drug solubility, the drug spreads only in one dimension, the substance particle is smaller than the size of a carrier, swelling of the system and its dissolution are insignificant, the drug diffusivity does not change, and sink conditions are achieved. The Higuchi relationship is generally used for transdermal systems and matrix tablets loaded with water soluble and low soluble drugs incorporated in semisolid and solid matrices. The matrix releases the solid drug by simultaneous penetration of the surrounding liquid, dissolution of the drug, and leaching out of the drug through interstitial channels or pores for the Higuchi model [48–50].

## 4. Conclusion

Within the scope of the study, it was observed that HTCC could be synthesized from chitosan and the synthesized HTCC was soluble in acidic, basic, and alkaline environments. Together with the PVA partner, the PVA:HTCC scaffold fibers could be electrospun. *C. officinalis* extract could be loaded onto electrospun wound dressings. In addition, the loaded *C. officinalis* extract was released from the dressing for approximately 55 h, and the release behavior was consistent with the Higuchi kinetic model.

## Figures and Tables

**Table 1:** Compositions and the codes of the produced scaffolds.

Sample code	PVA:HTCC (w/w)	% *C. officinalis* added to polymer solution (w:w)	Crosslinking process
PVA	100:0	0	Not crosslinked
PVA/HTCC	70:30	0	Not crosslinked
PVA/HTCC/CO	70:30	10	Not crosslinked
C-PVA	100:0	0	Crosslinked
C-PVA/HTCC	70:30	0	Crosslinked
C-PVA/HTCC/CO	70:30	10	Crosslinked

**Table 2:** Average, minimum, and maximum fiber diameters of the scaffolds.

Sample code	Average diameter (nm)	Standard deviation	Minimum diameter (nm)	Maximum diameter (nm)
PVA/HTCC	372.73	123.27	306.10	598.67
PVA/HTCC/CO	629.51	111.82	489.77	876.68
C-PVA/HTCC	456.62	83.99	367.96	627.70
C-PVA/HTCC/CO	311.35	52.98	246.79	420.27

**Table 3:** The tensile strength, elongation at break, and Young’s modulus values of the scaffolds.

Sample code	Tensile strength (MPa)	Elongation at break (%)	Young’s modulus (MPa)
PVA	3.018	45.56	0.243
PVA/HTCC	10.45	24.75	1.186
PVA/HTCC/CO	14.42	31.18	1.134
C-PVA	4.762	5.064	0.717
C-PVA/HTCC	8.332	23.52	0.442
C-PVA/HTCC/CO	10.07	65.06	0.189

**Table 4:** Kinetic parameters of the scaffolds calculated for different kinetic models.

Kinetic model	Kinetic parameters	Regression coefficient (R^2^)	t_0.5_ (min)
**Zero order Q = Q** **_0_** ** + K** **_0_** ** t**	0.0205 mg min^−1^	0.9023	720.54
**First order Log C** **_t_** ** = Log C** **_0_** ** – k t / 2.303**	0.0012 min^−1^	0.8868	675.66
**Hixson–Crowell Q** **_0_** **^1/3^** **– Q** **_t_** **^1/3^** ** = K** **_HC_** ** t**	0.001 mg^1/3^ min^−1^	0.9106	707.35
**Higuchi m** **_t_** ** = k** **_H_** ** t ** **^1/2^**	1.3959 mg min^−1/2^	0.9601	530.26
**Korsmeyer–Peppas M** **_t_** **/M** **_α_** ** = K t** **^n^**	K = 119.78 min^−n^ n = 18.636	0.9195	1104.73
**Best fit model: Higuchi**			
